# Identification and Characterization of PERK Activators by Phenotypic Screening and Their Effects on NRF2 Activation

**DOI:** 10.1371/journal.pone.0119738

**Published:** 2015-03-17

**Authors:** Wensheng Xie, Marie Pariollaud, William E. Wixted, Nilesh Chitnis, James Fornwald, Maggie Truong, Christina Pao, Yan Liu, Robert S. Ames, James Callahan, Roberto Solari, Yolanda Sanchez, Alan Diehl, Hu Li

**Affiliations:** 1 Department of Biological Sciences, Platform Technology and Science, GlaxoSmithKline, Collegeville, Pennsylvania, United States of America; 2 Stress & Repair Discovery Performance Unit, Respiratory Therapy Area, GlaxoSmithKline, King of Prussia, Pennsylvania, United States of America; 3 Hollings Cancer Center, Department of Biochemistry and Molecular Biology, Medical University of South Carolina, Charleston, South Carolina, United States of America; 4 Department of Sample Management Technologies, Platform Technology and Science, GlaxoSmithKline, Collegeville, Pennsylvania, United States of America; 5 Stress & Repair Discovery Performance Unit, Respiratory Therapy Area, GlaxoSmithKline, Stevenage, Hertfordshire, United Kingdom; University of Hong Kong, HONG KONG

## Abstract

Endoplasmic reticulum stress plays a critical role to restore the homeostasis of protein production in eukaryotic cells. This vital process is hence involved in many types of diseases including COPD. PERK, one branch in the ER stress signaling pathways, has been reported to activate NRF2 signaling pathway, a known protective response to COPD. Based on this scientific rationale, we aimed to identify PERK activators as a mechanism to achieve NRF2 activation. In this report, we describe a phenotypic screening assay to identify PERK activators. This assay measures phosphorylation of GFP-tagged eIF2α upon PERK activation via a cell-based LanthaScreen technology. To obtain a robust assay with sufficient signal to background and low variation, multiple parameters were optimized including GFP-tagged eIF2α BacMam concentration, cell density and serum concentration. The assay was validated by a tool compound, Thapsigargin, which induces phosphorylation of eIF2α. In our assay, this compound showed maximal signal window of approximately 2.5-fold with a pEC_50_ of 8.0, consistent with literature reports. To identify novel PERK activators through phosphorylation of eIF2α, a focused set of 8,400 compounds was screened in this assay at 10 µM. A number of hits were identified and validated. The molecular mechanisms for several selected hits were further characterized in terms of PERK activation and effects on PERK downstream components. Specificity of these compounds in activating PERK was demonstrated with a PERK specific inhibitor and in PERK knockout mouse embryonic fibroblast (MEF) cells. In addition, these hits showed NRF2-dependent anti-oxidant gene induction. In summary, our phenotypic screening assay is demonstrated to be able to identify PERK specific activators. The identified PERK activators could potentially be used as chemical probes to further investigate this pathway as well as the link between PERK activation and NRF2 pathway activation.

## Introduction

Chronic obstructive pulmonary disease (COPD) presents a complex pathogenesis and is characterized by lung cell damage, resulting in decline of lung function, lung destruction and eventually premature death. Cigarette smoke is a major risk factor for COPD because it generates a high concentration of reactive oxidant species, imposing oxidative stress, severe inflammation and damage to lung structural cells. It is believed that promoting lung cell health and survival is a promising strategy for COPD treatment. The oxidative stress response is a crucial system to detoxify a variety of pollutants, promoting cell survival and providing protection to tissues and organs. NRF2 (Nuclear factor E2 related factor 2) is a transcription factor and master regulator of the oxidative stress response system [1]. In response to oxidative and electrophilic stress, NRF2 is stabilized and translocated to the nucleus where it activates downstream phase II gene transcription (such as HO-1 and NQO-1), leading to its anti-oxidant, cytoprotective and detoxifying functions [2,3]. Due to this critical regulatory effect in cell survival, NRF2 regulation represents an attractive mechanism for drug discovery for several diseases, such as neurodegenerative diseases and COPD [4–6]. Direct targeting of NRF2 activation by small molecules has been well studied and several small molecules have been reported as potent NRF2 activators [7,8]. We also established cell-based methods for the purpose of identifying new NRF2 activators for COPD [9]. Given the attractive therapeutic potential of this target, there are compelling reasons for identifying NRF2 activators with additional novel mechanisms of action, ideal selectivity and drug safety profiles.

Endoplasmic reticulum (ER) stress is thought to contribute to the pathogenesis of COPD [10–12]. The ER is the cell organelle in which synthesis, modification and correct folding of secreted proteins occur through tightly regulated processes. Dysregulation of these processes such as in response to oxidative stress from cigarette smoke results in the accumulation of unfolded or misfolded proteins which leads to ER stress. ER stress is involved in cell growth, differentiation and apoptosis, therefore, it is potentially critical in the pathogenesis of multiple diseases, including COPD, cancer and diabetes [13]. Indeed, it was reported that cigarette smoke induced protein damage and triggered the ER stress response in human alveolar epithelial cells [14]. Regulated ER stress initiates a process to restore protein homeostasis, resulting in protective effects on stressed cells. This ER stress / unfolded protein response (UPR) process comprises three major signaling pathways: ATF6, IRE1 and PERK (PRK-like endoplasmic reticulum kinase). As reviewed in [10,15,16], under stress conditions, ATF6, IRE1 and PERK are released from their ER membrane-bound protein BiP to activate their downstream effectors. ATF6, IRE1 and PERK function through different pathways with a common goal of reducing protein load in cells with misfolded or malfolded proteins. ATF6 and IRE1 upregulate the expression of ER protein folding machinery and degrade mRNA level in stressed cells to reduce overall protein synthesis. Alternatively, PERK activation reduces protein translation through the phosphorylation of its substrate, the eukaryotic translation-initiation factor 2α (eIF2α), resulting in the attenuation of protein synthesis. PERK is a transmembrane kinase located in the ER membrane. Under stress conditions, PERK is released from its binding partner BiP and dimerizes to become an active kinase. Activated PERK phosphorylates eIF2α which subsequently transmits the signal to its downstream effectors such as the C/EBP-homologous protein (CHOP). Besides eIF2α, other proteins may be a substrate of active PERK. Indeed, it was reported that PERK activation also directly activates NRF2 and induces PERK dependent cell survival [17,18]. Based on this rationale and reported evidence of NRF2 activation by PERK, we aimed to identify small molecule PERK activators and investigate their effects on the regulation of NRF2 signal pathway. This can potentially lead to discovery of small molecules to increase cytoprotection as potential novel therapeutics for treatment of diseases such as COPD.

We developed a cell-based LanthaScreen assay that measures the phosphorylation of eIF2α as a surrogate assay to monitor activation of PERK. LanthaScreen is a time-resolved fluorescence energy transfer (TR-FRET) based technology designed to detect post-translational modifications of protein in cells. It uses a Tb conjugated antibody that binds to the analyte as the fluorescent donor and GFP tag on the analyte as the fluorescent acceptor. In our assay, GFP tagged-eIF2α was delivered to U-2 OS cells via BacMam technology. The Tb labeled anti-phosphorylated eIF2α antibody binds to phosphorylated GFP-eIF2α upon compound treatment. The close proximity of Tb (fluorescent donor) to GFP (fluorescent acceptor) produces increased TR-FRET signal, in the form of ratio of GFP intensity vs. the Tb intensity. We describe herein the critical aspects for the development and optimization of the assay. We also demonstrate that the assay is robust, sensitive and amenable for high throughput screening (HTS). A focused set of 8,400 compounds was screened using this assay, yielding a number of hits. These hits were further characterized in a battery of secondary assays using primary human and mouse cells. Several hits were confirmed to activate PERK as demonstrated through Western blotting analysis using PERK knockout-derived mouse embryo fibroblast (MEF) cells and a selective PERK inhibitor. More importantly, we also show that confirmed hits were able to activate NRF2 dependent Phase II gene expression (such as HO-1). The significance of these results will be discussed.

## Materials and Methods

### Cell culture and treatment

U-2 OS cells were cultured in DMEM/F12 with 10% Fetal Bovine Serum (FBS) to reach approximately 70% confluence and cryopreserved in 90% FBS and 10% DMSO. For BacMam transduction, frozen U-2 OS cells were washed and resuspended in culture medium for direct use as described in the LanthaScreen section. Transduced cells were then treated with tool compounds Thapsigargin (Tg, purchased from Sigma, St. Louis, MO), Tunicamycin (Tn, purchased from Sigma, St. Louis, MO) or screening compounds.

MEF cells were prepared in Dr. Alan Diehl’s lab from wild type and PERK knockout mice as described in [19]. Prior to compound treatment, frozen MEF cells at passage 10 were recovered and cultured overnight in tissue culture dishes in DMEM medium with high glucose supplemented with 1X non essential amino acid, 1 mM glutamine, 0.55 mM β-mercaptoethanol and 10% FBS. After overnight incubation, the cell culture medium was replaced with fresh DMEM/F12 (no FBS) containing compounds and cells were incubated further for different lengths of time as described in figure legends. After treatment, cells were washed once with cold PBS and collected for Western blotting.

Normal Human Bronchial Epithelial (NHBE) cells were purchased from Lonza (Walkersville, MD). Cells were cultured with Lonza’s Bronchial Epithelial Cell Growth Medium Bullet kit in 5% CO_2_ at 37°C. Subculturing was performed with a ReagentPack Subculture Kit from Lonza as recommended by the manufacturer.

NHBE cells (at passage 1) for siRNA transfection experiments were seeded at 5.0 x 10^4^ cells/well in 24-well plates and placed overnight in a 37°C, 5% CO_2_ incubator.

### BacMam generation and transduction

GFP-eIF2α BacMam was generated and titrated as described previously [20]. The construct of the BacMam was confirmed by sequencing the bacmid. The BacMam transduction was performed with cryopreserved U-2 OS cells. Briefly, frozen U-2 OS cells were thawed, washed once with DMEM/F12 (without FBS, without Phenol red). Cells were resuspended in the same medium to the desired cell density. BacMam viruses were then added to the cell solution to the desired final MOT (Multiplicity of Transduction = the transducible virus numbers/cell). Subsequently, the resulting cell-BacMam mixture was transferred to a culture flask for overnight incubation at 37°C and 5% CO_2_. For some experiments during assay development, cells were mixed with GFP-eIF2α BacMam and directly plated into 384 well plates for overnight incubation. This in-plate transduction method achieved similar results to the in-flask transduction method. Therefore, due to logistical difficulties to perform compound stamping to plates with cells seeded, the in-flask transduction method was optimized for compound screening.

The IRE1/XBP-1 splicing reporter gene was prepared based on the information of [21]. Briefly, the XBP-1 splicing reporter gene BacMam construct consists of 5’ end of the XBP-1 open reading frame (including the 26 nucleotide “intron”) followed by a stop codon and the open reading frame of firefly luciferase. When ER stress is induced, IRE1 is activated and it removes the 26 nucleotide “intron” from XBP-1 which shifts the frame to bypass the stop codon, yielding a functional XBP-1 – luciferase fusion protein.

### LanthaScreen assay

In the initial set of experiments where the purpose was to evaluate the GFP-eIF2α BacMam transduction efficiency, we used the in-plate transduction method as described below. U-2 OS cells were mixed with different concentrations of BacMam viruses as indicated, and plated into a white 384 well cell culture plate (Cat# 784080, Greiner Bio-One) for an overnight incubation at 37°C with 5% CO_2_. Compounds were dispensed to the cell culture plate with a HP digital dispenser (HP D300, Hewlett-Packard, Corvalis, OR) and incubated for a defined time point in a cell culture incubator. After treatment, the culture medium was drained onto a paper towel. The Tb labeled anti phospho eIF2α antibody (Life Technology, Cat# PV4815, Carlsbad, CA) was diluted in TR-FRET buffer (Life Technology, Cat# PV3574, Camarillo, CA) and added to each well. The diluted antibody solution was also supplemented with 0.5% Triton X-100, 1x PhosSTOP (Roche Applied Science, Indianapolis, IN) and 1x Protease inhibitor (Roche Applied Science, Indianapolis, IN). After 1 h incubation at RT, LanthaScreen signal was measured with an EnVision plate reader at the excitation wavelength 340 nm, the first emission wavelength 520 nm (GFP intensity), and the second emission wavelength 495 nm (Tb intensity). The LanthaScreen signal was calculated as the ratio of the GFP channel intensity vs. the Tb channel intensity.

In subsequent experiments, U-2 OS cells were cultured in flasks (“in-flask” transduction) in the presence of GFP-eIF2α BacMam for overnight. The transduced cells were trypsinized, washed and resuspended in DMEM/F12 to desired conditions (as indicated in figure legends). This cell solution was dispensed into white 384 well cell culture plates at 10 μL/well. The plates were pre-stamped with compounds with an Echo 555 dispenser (Labcyte Inc., Sunnyvale, CA). The plates with the mixture of cells and compounds were then incubated at 37°C and 5% CO_2_ for 2.5 h (or as indicated otherwise). Subsequently, the medium was removed with an EL406 plate washer (BioTek, Winooski, VT). The diluted Tb labeled anti phospho-eIF2α antibody solution (prepared as above) was added to the plate at 10 μL/well. The antibody solution was incubated with cells for 1 h at room temperature, or overnight at 4°C. LanthaScreen signal was measured as described above.

### Compound screening and pEC_50_ determination

A focused collection of GSK compounds containing 8,400 compounds was chosen for the screen and this set is also called “cell penetration and pathway set”. All compounds were tested in 384 well plate format. For single concentration screening, compounds were prepared in DMSO at 1 mM as stock solutions. For full curve screening, compounds were dissolved in DMSO at 10 mM. Serial dilution was performed in DMSO to generate appropriate concentrations. Compounds were prepared in master plates and were stamped at 100 nL to 384 well cell culture plates. The 384 well plates bearing the stamped compounds were then added with BacMam transduced U-2 OS cells at 10 μL/well at the desired cell density. In all 384-well screening plates, column 6 was added DMSO only as low control; column 18 was added Tg at a final assay concentration of 1 μμM as high control. For single dose screening, the compound activity was calculated as percent activation using the two controls: the percent activation = 100*(The sample LanthaScreen signal – the average of low control)/(the average of high control – the average of low control)%. A hit was defined when the compound showed above 20% activation of the signal in duplicate, and the compound had no intrinsic fluorescence interference. For pEC_50_ determination, the Graphpad Prism program with an activator non-linear equation was used to calculate the pEC_50_ value using the percent activation data derived from 11-point dose response curves.

### Western blotting reagents and methods

To prepare cell lysates for Western blot, MEF cells were lysed in Cell extraction buffer (Life Technologies, Cat# FNN0011), supplemented with 1x PhosSTOP and Protease inhibitors (the same products as used in the LanthaScreen assay part). NHBE cells were lysed in Roche complete lysis-M buffer (Cat#04719956001, Roche). Proteins were resolved by 10% or 12.5% SDS-PAGE and transferred to a membrane. Blots were probed with different primary antibodies as indicated in the figure legends. Rabbit Anti eIF2α phospho Ser52 antibody was from Upstate (Cat# 07–760, Lake Placid, NY, 1:1000 dilution), rabbit anti total eIF2α antibody and anti ATF-4 antibody were both from Cell Signaling Technology (Cat# 9722 and #1185, Danvers, MA, 1:1000 dilution). The secondary antibodies for eIF2α western blotting analysis were IRDye 680-conjugated goat anti rabbit IgG (Li-Cor Bioscience, 1:5000 dilution). The PERK antibody and secondary antibody were both from R&D Systems (Cat# AF3999 and #HAF017, Minneapolis, MN, 1 μg/mL and 1:2000 dilution, respectively). Rabbit anti-Heme oxygenase 1 (HO-1) polyclonal antibody was from Enzo Life Sciences (Cat# ADI-SPA-894, Farmingdale, NY, 1:2000 dilution). The secondary antibody was HRP-conjugated anti rabbit IgG (Cat# 7074, Cell signalling, 1:2000 dilution). Mouse monoclonal anti-GADD153/CHOP was from Santa Cruz Biotechnology (Cat# sc-7351, Dallas, TX, 1:250 dilution). The secondary antibody was HRP-conjugated anti mouse IgG from R&D system (Cat# HAF007, 1:1000 dilution). NQO-1 antibody was from Abcam (Cat. Ab34173). The control β-actin antibody was from Santa Cruz (Cat# sc-1616).

### XBP-1 splicing reporter luciferase analysis

A luciferase reporter gene construct was designed to include the XBP-1 splicing site which enables the reporter expression only under ER stress conditions. HT1080 fibrosarcoma cells were pretransduced with XBP-1 luciferase reporter BacMam. Frozen cells were then prepared after the transduction. For testing compound effects on IRE1/XBP-1 activity, frozen pretransduced HT1080 fibrosarcoma cells were thawed, washed and resuspended in EMEM media supplemented with 1% FBS. Cells were plated at 1.5x10^4^ cells/well in 384 well plates and treated with compounds for 3 h in a 37°C, 5% CO_2_ incubator. After the treatment, luciferase activity was measured with Steady-Glo luciferase reagents (Cat# E2550, Promega) according to the kit instruction.

### siRNA transfection reagents and methods

5X siRNA Buffer (Cat# B-002000-UB-100), DharmaFECT 1 Transfection Reagent (Cat# T-2001–02), NonTargeting siRNA (Cat# D-00180–10), PERK (Cat. L-004883–00), or NFE2L2 (Nrf2, Cat# L-003755–00) siRNAs were purchased from Thermo Scientific. OptiMem reduced serum medium was purchased from Life Technologies (Cat# 31985062).

The NHBE cells (at passage 1) were transfected with 25 nM PERK, NRF2 or non-targeting siRNAs using DharmaFECT 1 transfection agent according to manufacturer’s protocol. Cells were then incubated for 48 h prior to compound treatment. After the siRNA treatment, medium was replaced with fresh medium containing compounds or DMSO as described in the figure legends. Cell lysates were then prepared for the phospho and total eIF2α, HO-1, NQO1, CHOP protein or mRNA expression studies.

### NQO-1 ELISA

The antibodies and recombinant protein for the NQO-1 ELISA experiments were procured from Abcam. Mouse monoclonal anti-NQO-1 was used as the coating antibody (Cat# ab28947) while rabbit polyclonal anti-NQO-1 was used as the detection antibody (Cat# ab34173). Recombinant full-length human NQO-1 protein (Cat# ab59663) was used to generate the NQO-1 standard curve. NQO-1 ELISA was performed using common ELISA procedures. Briefly, plates were coated with mouse monoclonal anti-NQO-1 antibody. After overnight incubation, plates were blocked with 1% bovine serum albumin (BSA) in Phosphate Buffered Saline buffer (PBS). Cell lysates were incubated with coated plates for desired time. After the detection antibody incubation, tetramethylbenzidine (TMB) reagent (BD Biosciences) was added to the plates. Finally, the plates were read at 450 nm on a SpectraMax384 plate reader (Molecular Devices).

## Results

### Schematic of LanthaScreen Assay principle

**Fig 1:**
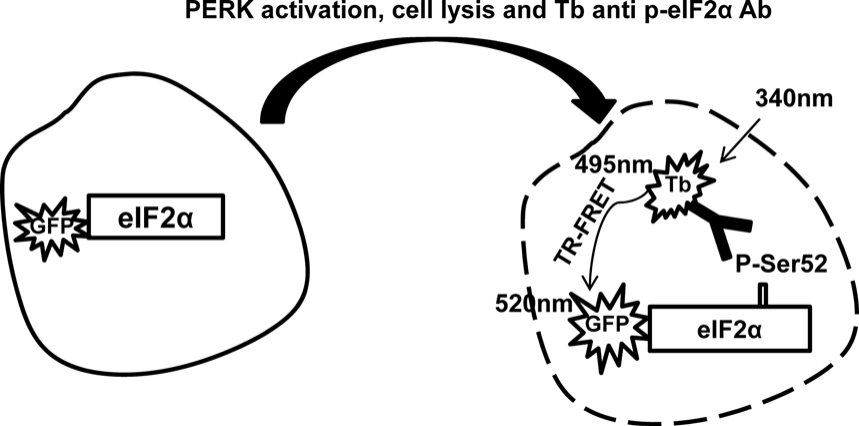
Scheme of PERK dependent eIF2α phosphorylation and the principle of LanthaScreen detection of p-eIF2α.

Fig. 1 illustrates the assay principle for detecting p-eIF2α in the cell lysate with the LanthaScreen technology. Cells are transduced with GFP-eIF2α BacMam and treated with PERK activators (such as Tg). The activation of PERK by PERK activators causes the phosphorylation of eIF2α. The Tb-labeled anti-phospho eIF2α antibody binds to GFP-eIF2α phospho Ser52. The close proximity between the donor Tb and the acceptor GFP enables the energy transfer from Tb to GFP, hence forms a FRET signal. More specifically, when Tb and GFP are in close distance, upon excitation at 320 nm, Tb exhibits 495 nm emission which is transferred to GFP. This energy transfer increases the emission of GFP at 520 nm. The ratio of GFP intensity over the Tb intensity (520 nm intensity/495 nm intensity, i.e. LanthaScreen TR-FRET signal) is directly proportional to the level of p-eIF2α in cell lysate.

### Assay optimization

**Fig 2:**
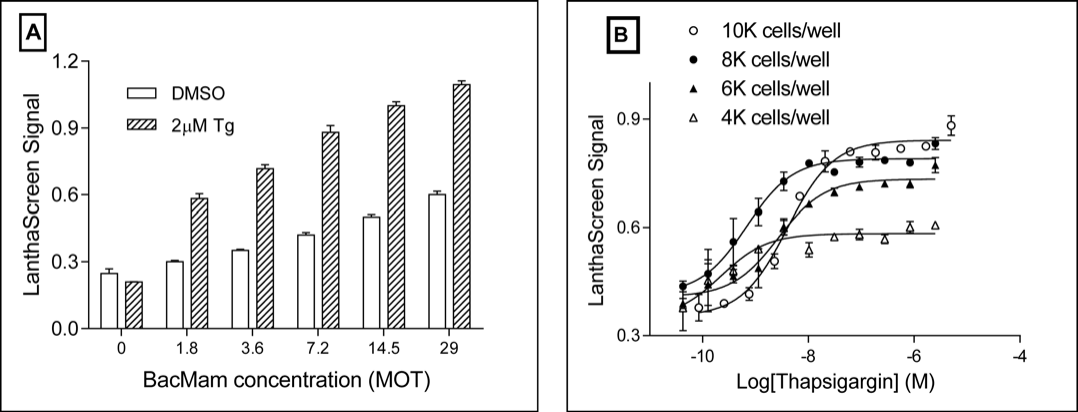
Titration of GFP-eIF2α BacMam. (A) U-2 OS cells were mixed with different amount of GFP-eIF2α BacMam and plated to a 384 well culture plate for overnight. Cells were then treated with either DMSO or 2 μM Tg for 1 h at 37°C 5% CO_2_. LanthaScreen was performed to detect eIF2α phosphorylation. Data was the average of 4 repeats. (B) U-2 OS cells were transduced with GFP-eIF2α BacMam in a culture flask with the optimized MOT for overnight. After trypsinized and washed, cells were resuspended to different cell density and plated into a 384 well plate. After the treatment with Tg for 2.5 h at 37°C and 5% CO_2_, LanthaScreen assay was performed to detect the phosphorylation of GFP-eIF2α. Data was the average of 3 repeats.

We first evaluated the GFP-eIF2α BacMam and the LanthaScreen technology in U-2 OS cells. The DNA construct for the BacMam generation was verified by sequencing. The expression of GFP was observed with a microscope in GFP-eIF2α BacMam transduced U-2 OS cells, but not in the control cells. Next, U-2 OS cells were mixed with different amounts of GFP-eIF2α BacMam from 0 to 29 MOT (virus particles / cell number) and plated directly into 384 well plates for overnight incubation. On the following day, the transduced cells were treated with either DMSO or the ER stress inducer such as Tg at a final concentration of 2 μM for 1 h at 37°C. The LanthaScreen was then performed to detect the phosphorylation of eIF2α. As shown in Fig. 2A, only in the presence of GFP-eIF2α BacMam, Tg treatment significantly increased the signal over DMSO control. The signal / background ratio (LanthaScreen signal with Tg / LanthaScreen signal with DMSO) was the highest at MOT 7.2. It was also noticeable that increasing BacMam concentration alone also caused increase of the LanthaScreen signal. This indicated that BacMam transduction by itself generated a certain level of stress to cells and caused some level of eIF2α phosphorylation. In light of this observation, we selected the lowest BacMam concentration which was still able to generate the required signal / background ratio for the screening.

The data above demonstrated the feasibility of developing a high throughput LanthaScreen assay. The aforementioned in-plate transduction format would work for testing a small set of compounds. However, it would present a huge logistical challenge for dispensing a large number of compounds directly to plates with cells seeded. We therefore explored an in-flask transduction format. In this format, U-2 OS cells were transduced in 250 mL culture flasks for 16 h, trypsinized and plated into 384 well plates which were pre-stamped with screening compounds. Based on the in-plate transduction results of BacMam concentration, we retitrated the BacMam concentration in a small range from MOT of 2.6 to 10.4 in the in-flask transduction format. Similar results were obtained with the in-plate transduction format, where BacMam concentration of MOT of 5.2 produced the optimal signal / background ratio (data not shown). We decided to use this BacMam concentration for the overnight in-flask transduction for the following experiments.

Cell density is critical for compound potency and assay signal. To obtain an optimal assay signal, we titrated the cell density during compound treatment step in 384 well plates. After overnight BacMam transduction, cells were trypsinized, washed and resuspended to density at 400 cells/μL, 600 cells/μL, 800 cells/μL and 1,000 cells/μL, respectively. Cells were added to 384 well cell culture plates at 10 μL/well and treated with tool compounds Tg and tunicamycin (Tn) at different concentrations. After 2.5 h incubation at 37°C and 5% CO_2_, LanthaScreen assay was performed to detect eIF2α phosphorylation level. As shown in Fig. 2B, the condition with 4,000 cells/well showed a very low signal increase under the treatment of either Tg or Tn. The condition of 6,000 cells/well had a higher signal increase than 4,000 cells/well. However, 8,000 cells/well had even higher signal increase. For the condition of 10,000 cells/well, although the signal increase was the highest, the potency of both Tg and Tn presented a right shift (lower potency). HTS assay often requires not only high signal / background ratio to ensure the robustness, but also the sensitivity to measure compound potency. We hence chose 8,000 cells/well for the final cell density during the compound treatment.

Following a similar principle, we further optimized serum concentration, antibody concentration and treatment time. For the sake of simplicity of the manuscript, optimization details and data are not described here. When all the optimization steps were completed, we obtained the assay conditions suitable for compound screening as below.

### PERK activity dependency of assay signal

**Fig 3:**
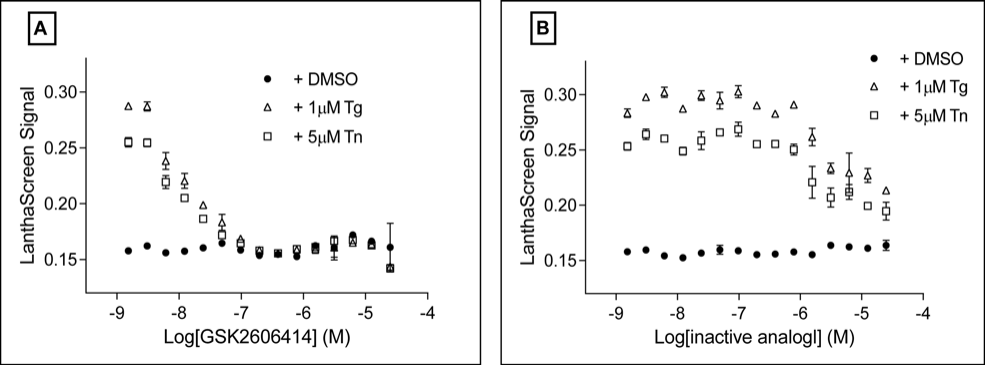
LanthaScreen assay signal was PERK dependent. U-2 OS cells were transduced with GFP-eIF2α BacMam under the optimized conditions for overnight. Cells were then trypsinized and plated into a 384 well plate. Different doses of a specific PERK inhibitor GSK2606414 (A) or an inactive analog (B) was incubated with cells for 30 min at 37°C and 5% CO_2_. DMSO, 1 μM Tg or 5 μM Tn was then added and incubated for 2 h at 37°C 5% CO_2_. Phosphorylation of GFP-eIF2α was analyzed with LanthaScreen assay. Data was the average of 2 repeats.

Before screening a focused compound library, we validated the assay specificity with a specific PERK inhibitor. Transduced U-2 OS cells were incubated with a PERK specific inhibitor GSK2606414 [22] and a structurally related but inactive analog [23] at different doses for 30 min. Tg or Tn were then added to a final concentration of 1 μM or 5 μM, respectively. After further 2 h incubation at 37°C, LanthaScreen was performed to detect eIF2α phosphorylation. As shown in Fig. 3A, the PERK inhibitor itself did not affect the LanthaScreen signal, nor did the negative control (inactive compound) (Fig. 3B). In contrast, both Tg and Tn gave more than 2 fold signal increase, which was completely inhibited by the PERK specific inhibitor at concentration 10 nM or above. The inactive analog only mildly reduced the assay signal at concentration above 5 μM. Clearly, these data demonstrated that phosphorylation of GFP-eIF2 α in the assay was dependent on PERK activity.

### Results from the focused screening

After optimizing all the assay conditions as described above, we automated the plate wash steps using an EL406 plate washer to achieve higher throughput. The automated assay now had the required robustness and the throughput for screening a large number of compounds. In 384 well screening plates, we added neat DMSO to column 6 as low control (0% activity) and 1 μM Tg to column 18 as high control (100% activity). We then screened the “cell pathway and penetration set” of 8,400 compounds at a single dose of 10 μM. Table 1 summarizes the statistics of the primary screening. The average Z’ is above 0.6 with signal / background ratio above 2.0. The cutoff value was set at 20% which was slightly above 3 standard deviations. This generated approximately 7% hit rate, which was considered in the acceptable range for this focused set. Compounds were selected as hits only when they showed above 20% response in duplicates.

**Table 1:** Robustness features of the LanthaScreen assay.

	Number of plates	Low control signal	High control signal	S/B	Z’
**1** ^**st**^ **screen**	15	0.17	0.404	2.4	0.74
**2** ^**nd**^ **screen**	25	0.15	0.37	2.4	0.8
**3** ^**rd**^ **screen**	4	0.23	0.48	2.1	0.62

U-2 OS cells were transduced with GFP-eIF2α BacMam for overnight in flasks. Cells were then trypsinized, washed and resuspended for plating into 384 well plates for compound treatment. The low control was DMSO only, the high control was 1 μM Tg. All the conditions were the optimized final conditions as described in results.

**Fig 4:**
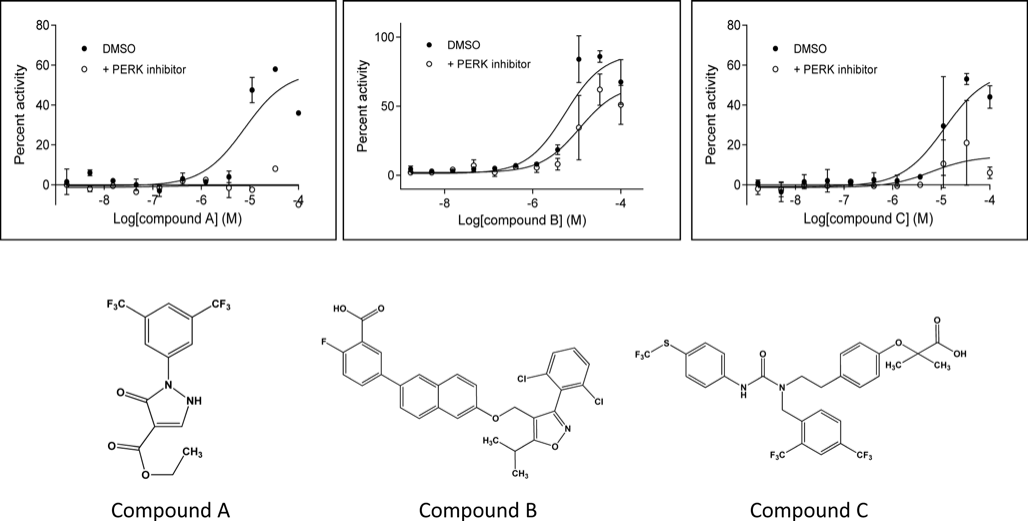
Dose curves and structures of 3 compounds from focus screening. For the activity analysis, GFP-eIF2α BacMam transduced U-2 OS cells were incubated with DMSO or 0.5 μM PERK inhibitor for 30 min at 37°C and 5% CO_2_, then plated into 384 well plates and treated with compounds at different doses for 2.5 h at 37°C and 5% CO_2_. Cells treated with 2 μM Tg only were used as the control for 100% activity. LanthaScreen was performed for detection of phospho-GFP-eIF2α. Data was the average of 2 repeats.

Based on the above statistical cutoff, 190 compounds were selected as hits and were followed up with dose-response confirmation. Meanwhile, based on comparison of fluorescence intensity, compounds with high fluorescence intensity were removed. We identified 42 compounds which showed good dose response curves without fluorescence interference. We further characterized these 42 compounds by analyzing their PERK dependent activity using the specific PERK inhibitor. Similar to what described above, transduced U-2 OS cells were incubated with DMSO or with the PERK inhibitor first, then cells were incubated with these hits at different doses. Data from this experiment showed that several compounds had relatively good dose curve responses and PERK dependent activities. Fig. 4 shows the dose-response curves of 3 representative compounds in the absence or presence of PERK inhibitor. The percent activation was expressed in comparison to the activity of the high control 2 μM Tg (considered as 100% activation). Thus, compound A (ethyl 2-(3,5-bis(trifluoromethyl)phenyl)-3-oxo-2,3-dihydro-1H-pyrazole-4-carboxylate) showed approximately 60% activation and this activation was almost completely inhibited by the PERK inhibitor, strongly suggesting that the activity of this compound was PERK dependent. Compound B (5-(6-((3-(2,6-dichlorophenyl)-5-isopropylisoxazol-4-yl)methoxy)naphthalen-2-yl)-2-fluorobenzoic acid) induced about 90% activation at the highest doses. The activity decreased to around 50% in the presence of the PERK inhibitor, indicating a portion of its activity was PERK dependent. Compound C (2-(4-(2-(1-(2,4-bis(trifluoromethyl)benzyl)-3-(4-((trifluoromethyl)thio)phenyl)ureido)ethyl)phenoxy)-2-methylpropanoic acid) induced around 50% activation at high doses, and dropped to around 20% in the presence of the PERK inhibitor. The structures of these three compounds are shown in Fig. 4. Further characterization of these three compounds such as the effects on the PERK downstream pathway and NRF2 dependent Phase II gene expression in different cell types was carried out and is described below.

### Identified activators induced PERK signaling pathway components in MEF cells and NHBE cells

**Fig 5:**
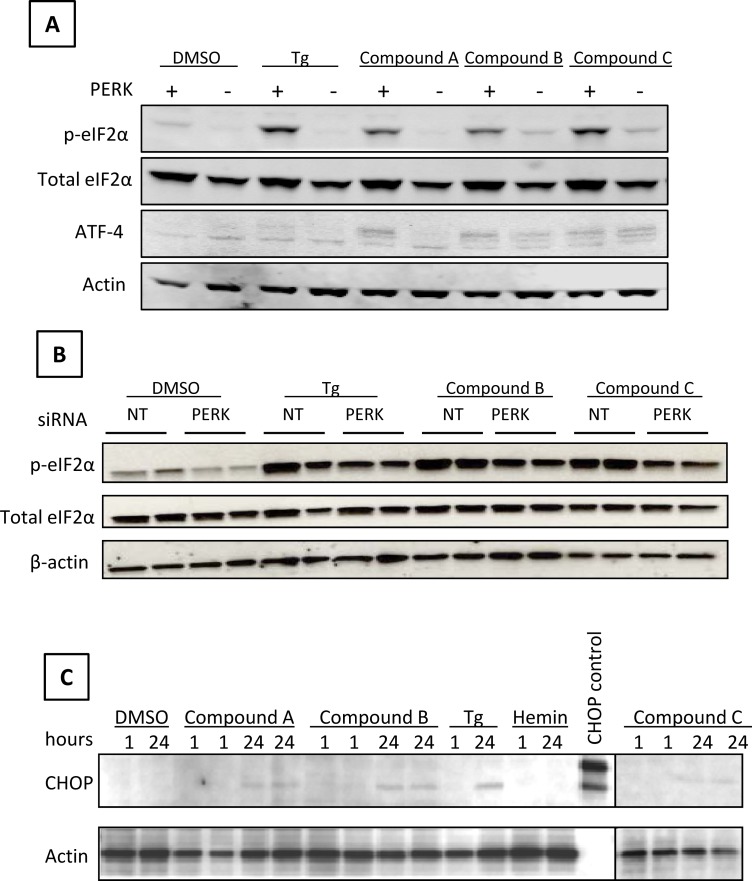
Compounds induce PERK downstream signaling. (A) Wild type and PERK knock out MEF cells were treated with 1 μM Tg or 20 μM compounds for 1 h. (B) NHBE cells were transfected with PERK siRNA or non-targeting (NT) siRNA for 48 h, then treated with compounds at 3 μM for 24 h. (C) NHBE cells were treated with compounds at 10 μM for 1 h or 3 μM for 24 h. Tg (0.5 μM) and hemin (3 μM) served as controls. Treatment was all at 37°C and 5% CO_2_. Cells were lysed and protein expression was determined by Western Blotting. Data was representative of at least 2 separate experiments.

As described above, several compounds were identified through the focused screening using the developed LanthaScreen method. In addition, we demonstrated PERK dependent eIF2α phosphorylation by these compounds using a specific PERK inhibitor. To confirm the specificity of these compounds, we compared the activity of these activators in wild type and PERK knockout-derived MEF cells. As shown in Fig. 5A, treatment with Tg produced a dramatic increase in p-eIF2α in the wild type MEF cells, but not in the PERK knockout MEF cells. Compound A also showed significant induction of p-eIF2α in the wild type MEF cells, but not in the PERK knockout MEF cells. These data are not only consistent with the LanthaScreen data, but also strongly suggest PERK dependent activity of compound A. Compounds B and C showed good induction in the wild type MEF cells and modest induction in the PERK knockout MEF cells, suggesting that their activities are also significantly dependent on PERK but with a portion from non-PERK kinase(s). ATF-4, a downstream component of PERK signaling pathway, was also clearly induced by these three compounds in wild type MEF cells. Compound A did not generate detectable ATF-4 signal in PERK knockout MEF cells, whereas compounds B and C had clear increase on ATF-4 signal in PERK knockout MEF cells. This was consistent with their effects on eIF2α phosphorylation. It again supported the hypothesis that the activity of compound A was mainly PERK dependent, while compounds B and C had a small portion of non-PERK dependent activity.

In order to demonstrate that these compounds show similar activity in human cells, we examined the activity of compound B and compound C in Normal Human Bronchial Epithelial (NHBE) cells using RNA interference. NHBE cells were transiently transfected with PERK-specific and non-targeting siRNAs prior to compound treatment. Fig. 5B shows that these compounds increased phosphorylation of eIF2α in NHBE cells under the non-targeting siRNA condition. More importantly, when PERK expression was knocked down by specific siRNA (PERK protein knockdown was confirmed by Western blot as demonstrated in the next experiment), the induction of eIF2α phosphorylation was significantly lower than in the non-targeting siRNA control. This further demonstrated that the phosphorylation of eIF2α by these compounds was PERK dependent in NHBE cells. Due to insufficient supply of compound A, its activity was not tested in NHBE cells.

To further interrogate the downstream effects of these potential PERK activators in NHBE cells, the expression of CHOP protein, an important downstream component of PERK signaling pathway, was determined. As shown in Fig. 5C, after 1 h treatment, CHOP protein level was undetectable under all experimental conditions. After 24 h treatment, all three compounds clearly increased the level of CHOP protein. Compound B seemed to be relatively more effective on the induction of CHOP protein level.

**Fig 6:**
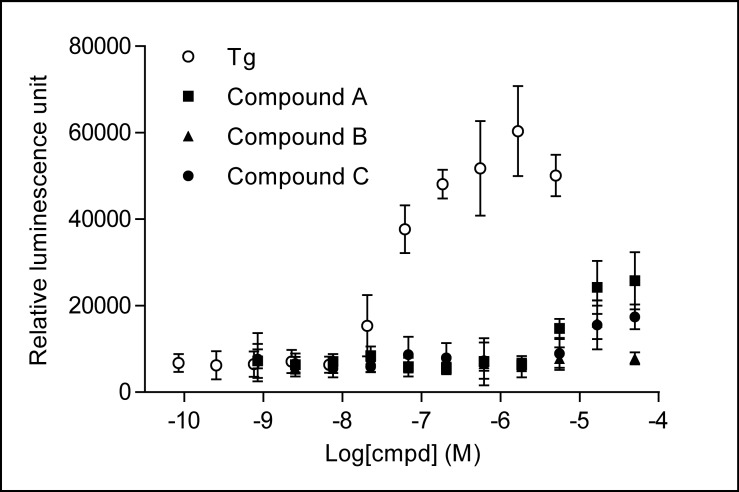
Compounds have insignificant activity in IRE1 branch. HT1080 cells pretransduced with XBP-1 splicing reporter gene were treated with compounds at different concentrations for 3 h at 37°C and 5% CO_2_. Luciferase activity was measured with Steady Glo reagents after the treatment. Data were average of 8 repeats.

We also tested the effects of these compounds on IRE1 branch by using a XBP-1 splicing reporter gene. In this reporter gene construct, a stop codon is added in N terminus to the luciferase sequence. Under the normal non-stressed condition, the reporter gene is silent. In response to ER stress, IRE1 mediates an open reading frame shift and the luciferase reporter gene is expressed. This reporter system was validated by using Tg to mimic stress condition. As expected, Tg treatment generated a very strong dose dependent luciferase activity as shown in Fig. 6. This demonstrated that the reporter gene construct had the correct stress response to turn on and off the reporter gene. In comparison, compound A generated a very mild luciferase activity at the top two concentrations. Compounds B and C almost had only basal activity, indicating no or very little effect on IRE1 branch.

### PERK activators induced NRF2 dependent Phase II gene expression

**Fig 7:**
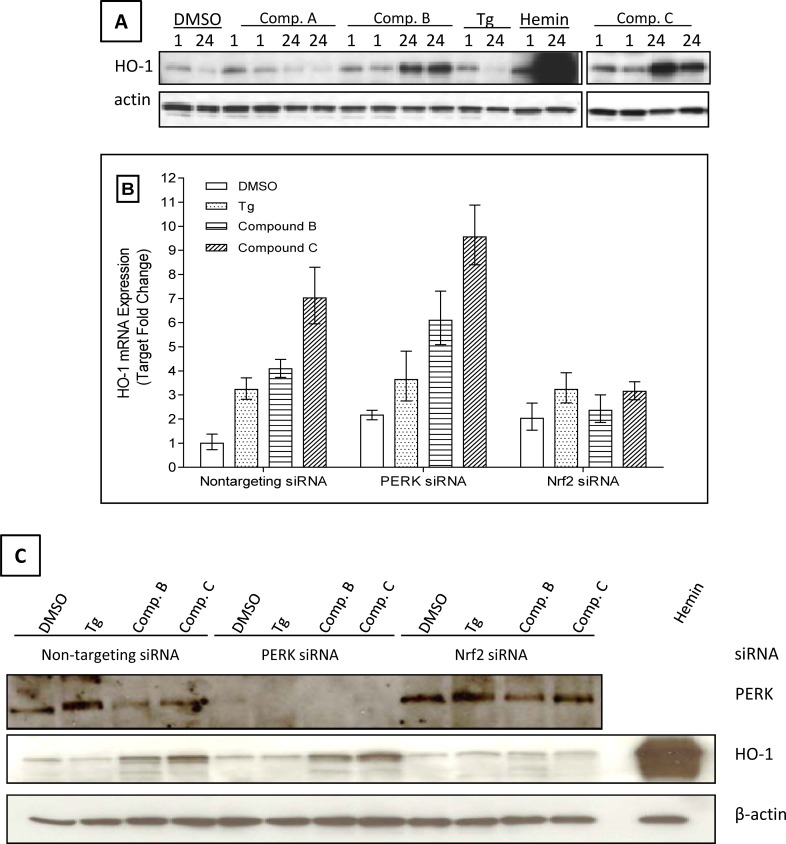
Compounds increase Heme Oxygenase-1 (HO-1) expression in a NRF2 dependent pattern. (A) NHBE cells were treated with 10 μM of compound for 1 h or 3 μM of compound for 24 h at 37°C and 5% CO_2_, respectively. Tg (0.5 μM) and hemin (3 μM) served as controls. HO-1 protein was then determined. (B) NHBE cells were transfected with 25 nM non-target siRNA, PERK siRNA or NRF2 siRNA for 48 h, then treated with compounds at 3 μM for 24 h. HO-1 mRNA level was then analyzed. (C) NHBE cells transfected with non-targeting, NRF2 or PERK siRNA for 48 h, treated with DMSO, 0.5 μM Tg, 3 μM compound B, C or hemin for 24 h. HO-1 protein level was determined. Data was representative of 3 experiments.

As described above, the identified PERK activators showed PERK dependent signaling effects in MEF and NHBE cells. To determine if these compounds activate the NRF2 pathway, we measured induction of HO-1 and NQO-1 proteins, two archetypical NRF2 modulated genes, by these compounds. As shown in Fig. 7A, both compound B and compound C showed high induction of HO-1 protein after 24 h treatment. In contrast, compound A showed mild induction after 1 h treatment, and a decreased signal after 24 h treatment. The decreased signal might be due to cytotoxicity after 24 h treatment as the band intensity of loading control actin was also noticeably lower. Hemin, a known HO-1 inducer used as a positive control, dramatically induced HO-1 protein level after 24 h treatment. Alternatively, Tg did not induce the expression of HO-1 protein after 24 h treatment. To confirm if these compounds affect the transcriptional level, HO-1 mRNA level was also analyzed in NHBE cells. Together with the purpose to check the NRF2 dependency of HO-1 expression, we performed siRNA transfection in NHBE cells before the compound treatment. As shown in Fig. 7B, the HO-1 mRNA was significantly increased with the treatment of Tg, compound B, or C under the condition of non-targeting siRNA (first panel). More importantly, we demonstrated that the activation of HO-1 was NRF2 dependent as shown in the NRF2 siRNA treatment. When NHBE cells were treated with NRF2 siRNA, the induction of HO-1 mRNA was diminished. Interestingly, PERK siRNA treatment did not reduce the increase of HO-1 mRNA by the compounds. As shown in Fig. 7A, PERK protein was undetectable after PERK siRNA treatment, confirming PERK siRNA knockdown effect. Consistent to the mRNA level change, the NRF2 dependent HO-1 upregulation was also observed at the protein level. As shown in Fig. 7C, under non-targeting siRNA condition, both compounds B and C induced HO-1 protein level. This increase was diminished under the condition of NRF2 siRNA treatment. Again, PERK siRNA treatment did not significantly affect the induction of these two compounds on HO-1. In these experiments, we also consistently noticed that Tg did not affect the protein level of HO-1, but it significantly induced the HO-1 mRNA level.

**Fig 8:**
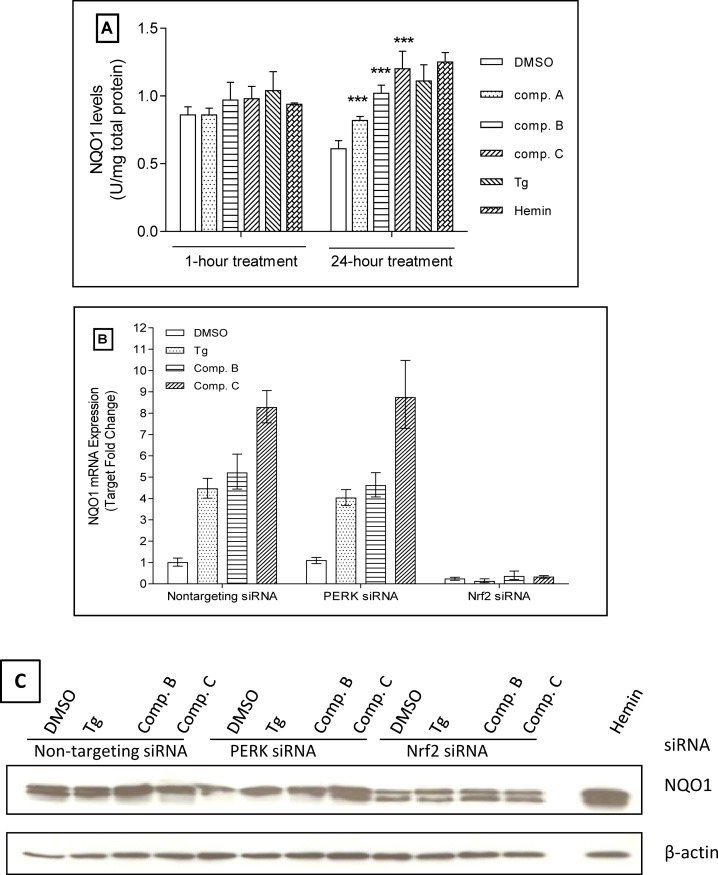
Compounds increase NQO-1 protein expression in a NRF2 dependent pattern. (A) NHBE cells were treated with 10 μM compound for 1 h or 3 μM compound for 24 h at 37°C and 5% CO_2_. NQO-1 protein level was determined by ELISA. Stars indicate p value ≤0.001 (n = 3). (B) NHBE cells were transfected 25nM siRNA for 48 h, treated with DMSO, 0.5μTg, 3 μM compound B or C for 24 h. NQO-1 mRNA level was then determined by Real-Time PCR and was normalized with DMSO control (n = 3). (C) NHBEs were transfected 25 nM siRNA for 48 h, treated with DMSO, 0.5 μM Tg, 3 μM compound B or C for 24 h. NQO-1 protein level was determined by Western blot. Data was representative of 3 experiments.

NQO-1 gene expression was analyzed in a similar manner. As shown in Fig. 8A, after 24 h treatment, Tg and Hemin doubled the protein signal. Our PERK activators also significantly augmented NQO-1 protein level after 24 h treatment, particularly with compounds B and C. The 1 h treatment with the two compounds did not significantly change the signal. This is likely due to insufficient amount of time for protein expression. More importantly, we also demonstrated that the induction of NQO-1 was NRF2 dependent through siRNA knockdown experiments. Similar to the HO-1 experiments, both the mRNA level and protein level of NQO-1 were analyzed with RT-PCR or Western blot, respectively. As shown in Fig. 8B and C, under the condition of non-targeting siRNA, compounds B and C induced NQO-1 expression. In contrast, under the condition of NRF2 siRNA, the increase of NQO-1 expression by these two compounds was almost diminished.

## Discussion

### Development of LanthaScreen for compound screening

The LanthaScreen assay was optimized with respect to several critical parameters: concentration of BacMam used in transduction, cell density, serum concentration and compound treatment time. BacMam concentration significantly affected the GFP-eIF2α expression level, which was important to determine robust signal / background ratio. On the other hand, higher BacMam concentration resulted in higher basal level of eIF2α phosphorylation and hence lower signal / background ratio, raising the possibility that the transduction itself at high level might generate some toxicity or mild stress on cells. Because the stress signaling pathway constitutes the basis for the readout of the assay, we wanted to minimize the stress responses introduced by the BacMam transduction. Therefore, we chose the lowest BacMam concentration able to generate sufficient signal / background ratio (approximately 2:1) to produce a suitable screening assay. Similarly, cell density and serum concentration were titrated to further optimize signal / background ratio. To validate the assay, two positive controls, Tg and Tn, were tested in the final conditions of the optimized assay. The estimated potency of Tg and Tn obtained from dose-dependent curves in the assay was comparable to that of literature data [24], demonstrating the feasibility of the approach. The developed LanthaScreen assay also exhibited robustness features suitable for high throughput screening as shown in Table 1. This was further validated by the success of the screening with a focused set of 8,400 compounds under a single concentration in duplicate. The initial hits from the duplicated single concentration screening were further analyzed in 11-point dose curves and fluorescence interference. Three compounds were eventually selected for further analysis to confirm their on-target specificity. Through the usage of PERK knockout MEF cells, we were able to confirm that the induction of eIf2α phosphorylation by compounds A and C was highly PERK dependent, while the activity of compound B was less PERK dependent. Indeed, the Western blot results generated with PERK knockout or wild type MEF cells were consistent with the LanthaScreen data.

Besides PERK activation, eIF2α phosphorylation can be regulated through other kinases such as PKR and GCN2 [19]. It is therefore important to ensure the detected signal is through PERK activation. We employed a potent and specific PERK inhibitor, together with a structurally related inactive analog, to test the specificity. Data showed that the assay signal induced by Tg or Tn, was reduced by the PERK specific inhibitor in a dose dependent manner. This strongly suggested that the LanthaScreen assay signal was mainly dependent on the activity of PERK. Moreover, we also tested several PKR inhibitors which showed significantly lower reduction to the response of Tg and Tn (data not shown). This implied that PKR contribution in the assay signal was minor, further suggesting that the detected signal was mainly PERK dependent. However, due to the complexity of the biological pathways implicated in these responses, secondary assay(s) are always necessary to confirm the on-target specificity of screened hits as discussed below.

### Downstream functional analysis of identified PERK activators

After the identification of these potential PERK activators, one important question to ask is whether they are able to influence PERK dependent downstream effects. The immediate target of activated PERK is eIF2α. Our data unequivocally demonstrated an elevated phosphorylation level of eIF2α with the treatment of the identified compounds both in MEF cells and NHBE cells. More importantly, the elevated phosphorylation level of eIF2α is PERK dependent, which is corroborated by data from PERK knockout MEF cells and PERK siRNA knockdown in NHBE cells. We also checked two further downstream components ATF-4 and CHOP which are critical for the ER stress induced apoptosis. As shown in Fig. 5A, compound A had a PERK dependent ATF-4 signal increase. Both compounds B and C also increased ATF-4 protein level in the wild type MEF cells, but they had detectable ATF-4 signal in the PERK knockout MEF cells as well. This suggested that compounds B and C had a small portion of non-PERK dependent activity. The increase of ATF4 protein level upon PERK activation is consistent with the notion that ATF4 and NRF2 both contribute and coordinate activation of anti-oxidation genes during ER stress response [25, 26]. In addition, as shown **in** Fig. 5C, our data demonstrated that the identified compounds induced the protein level of CHOP in NHBE cells after 24 h treatment.

Besides the PERK signaling pathway, ER stress has two other pathways, IRE1/XBP-1 and ATF6. Taking advantage of the XBP-1 activity assay developed in our group, we analyzed these compounds for IRE1/XBP-1 activity. Our data indicated that these compounds had mild activity on IRE1 branch at the tested top dose 50 μM. Compared to their effects on PERK branch, their effects of these compounds on IRE1 are much weaker. At this stage, we do not know whether these compounds induce effects on ATF6 signaling, which will be an interesting question for future studies.

Combining all the data of LanthaScreen assay and the protein detection in MEF cells and NHBE cells, we observed that all of the changes were overall correlated to the measured PERK activity and specificity. For example, in Fig. 4, compound A showed very good activity which was significantly inhibited by the PERK specific inhibitor. Consistently, in Fig. 5A, compound A induced strong eIF2α phosphorylation and ATF-4 protein in wild type MEF cells, but not in PERK knockout MEF cells. Moreover, compound A also induced CHOP protein level in NHBE cells with 24 h treatment (Fig. 5C). Taken together, these data demonstrate that the identified compounds are able to activate PERK and induce PERK dependent downstream signaling effects.

Here we would like to discuss two aspects which can be of interest to drug discovery and development. As we pointed out above, compounds B and C had small portion of non-PERK dependent activity. This small non-PERK dependent activity may provide some benefits to avoid adverse apoptotic effect from profound and chronic PERK activation. Mechanistically, the multiple biological effects may complicate target-focused investigation. However, for drug discovery, particularly with a phenotypic screening approach, any compound can be of interest if it demonstrates desired beneficial effects.

It is also very important to note the effect of different treatment conditions, particularly treatment time. Under the experiment conditions of Fig. 5A, CHOP protein level was undetectable in MEF cells with 3 h treatment. In NHBE cells, CHOP protein was not detectable with 1 h treatment, but clearly increased with 24 h compound treatment (as shown **in** Fig. 5C). The short time treatment (up to 3 h) did not increase CHOP protein level, which can be a critical point for choosing treatment conditions to avoid adverse effect of CHOP activation (see more discussion below).

### NRF2 pathway effects and therapeutic indications of identified PERK activators

**Fig 9:**
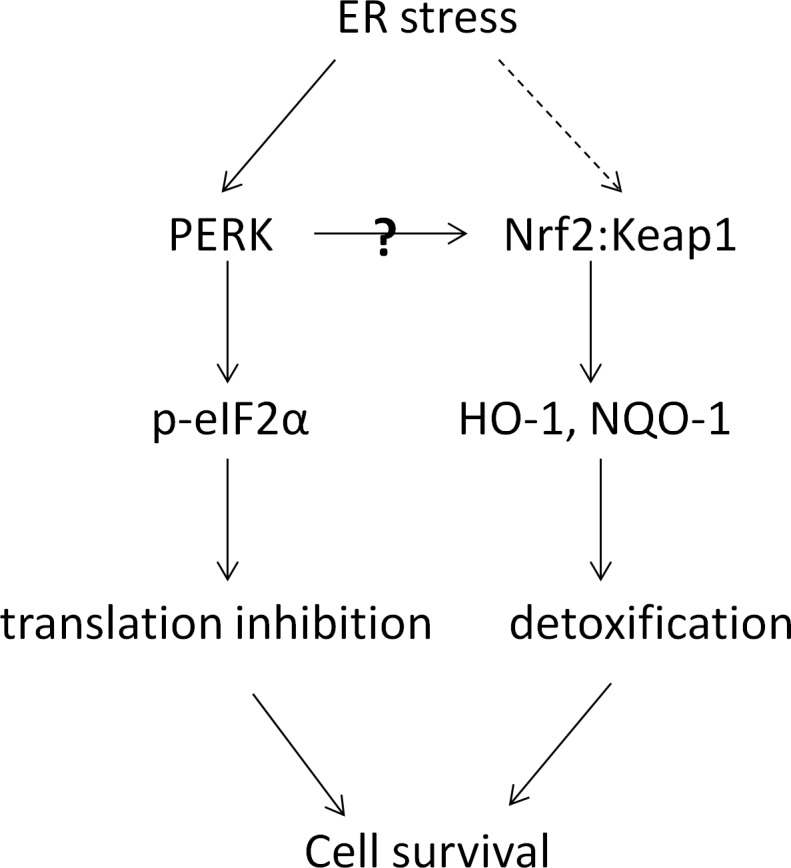
Model for potential crosstalk between PERK and NRF2 signaling pathways.

One interesting and yet novel hypothesis that we would like to explore further is the connection between PERK signaling pathway and the NRF2 oxidative stress response signaling pathway. Regulated activation of NRF2 pathway provides cytoprotection against oxidative or electrophilic insults. We examined the effects of our PERK activators on the expression of HO-1 and NQO-1, two key components of the NRF2-dependent defense system. Data demonstrated that these PERK activator compounds induced the expression of both HO-1 and NQO-1 in NHBE cells, the expression of which is NRF2 dependent. This strongly indicates that the compounds are able to modulate the activity of the important transcription factor NRF2 through a potential crosstalk between PERK and NRF2 signaling pathways, as shown in the proposed model (Fig. 9). Indeed, as shown in Figs. 7 and 8, NRF2 siRNA treatment prevented the compound induction on HO-1 and NQO-1, demonstrating that the effect is ultimately NRF2 dependent. These observations are consistent with our initial hypothesis that PERK signaling activation is able to augment NRF2 dependent signaling, with the potential of reducing cell damage and promoting cell growth in COPD lungs. We also noticed that PERK siRNA treatment did not significantly reduce the effect of compounds on HO-1 or NQO-1 expression (Figs. 7 and 8). One possible explanation is that our compounds had some non-PERK dependent activation on HO-1 and NQO-1 expression. This is possible since NRF2 regulation is complexed and related to a number of signaling pathways, such as JNK, PKC and ERK among others. Indeed, both compounds B and C showed some portion of non-PERK dependent activation on eIF2α and ATF-4. Alternatively, PERK siRNA treatment might have generated some stress responses which could stimulate HO-1 and NQO-1 expression. A third possibility is that our compounds activated the very small amount of PERK remained after siRNA treatment, and further upregulated HO-1 and NQO-1 expression, though this is less likely since our data showed a very effective knockdown of PERK protein. In addition, we observed similar results even after combining PERK siRNA treatment and a PERK inhibitor (data not shown). Nevertheless, our data indicates that these PERK activators interacted with NRF2 signaling pathway in our system. Although it is unclear whether the induction of HO-1 and NQO-1 by these compounds is directly PERK dependent, the connection between PERK activation and NRF2 activation is demonstrated. As proposed in Fig. 9, both PERK activation and NRF2 activation can lead to cell survival and provide cytoprotection. Based on this shared outcome, connections between these two pathways can be physiologically meaningful in terms of restoring the oxidant/antioxidant balance in COPD patients. It will be very interesting to further explore these connections. Another interesting observation is the effect of Tg on HO-1 and NQO-1 expression. Our data showed that Tg treatment significantly induced the mRNA level of both HO-1 and NQO-1, but not at the protein level (Figs. 7C and 8C). It is possible that the stability of HO-1 and NQO-1 proteins is regulated through some other cellular processes which may be differentiated from the mRNA regulation.

Taken together, we demonstrate here that our compounds, identified through a PERK activity dependent cell based assay, imposed PERK activation and were able to mediate NRF2 activation. Data also indicate that short term treatment of cells (even within 1 h) is sufficient to observe the activation of NRF2 pathway. Within this short treatment, we did not observe any significant cell death, which is supported by the equal amount of control protein β-actin. Under bright field microscopy, treated cells all appeared normal and healthy. Furthermore, CHOP protein was not induced until 24 h treatment in our experiment (Fig. 5C). It appears that an optimal treatment condition could be established to provide protective NRF2 activation yet to avoid an adverse pro-apoptotic effect from chronic PERK activation. Though an initial UPR is protective for stressed cells, chronic and unresolved UPR could lead to inflammation, apoptosis and cell death. It was actually reported that aberrant proteostasis was correlated with the severity of emphysema [27]. The authors of [27] proposed the ER stress inhibitor salubrinal as potential therapeutics. It is known that long term activation of ER stress signaling pathways can cause a series of disease responses. However, it is not uncommon that one signaling pathway can play different, even opposite biological effects under different physiological conditions. From drug discovery point of view, the key is to identify the proper compounds as well as the right treatment conditions. As demonstrated above, with appropriate treatment conditions (such as doses and time), we believe the adverse effect, if any, can be minimized while an optimal protective effect can be achieved. It is also interesting to note that compounds B and C have some non-PERK dependent activity but still show good NRF2 activation. This non-PERK dependent activity, though not purposefully developed from our hypothesis, may provide some mechanism to avoid the adverse effect from robust PERK activation. We are aware that the balance between the protective and damage effects of PERK and NRF2 pathways is somewhat intriguing but complexed. The crosstalk and interaction between these two pathways need to be further investigated. The *in vivo* biological effects of PERK activation on COPD pathology require more fundamental studies. Similarly, the developability of our compounds as therapeutic agents is beyond the scope of our current efforts.

Because of its impacts on cell survival / cell death, ER stress signaling pathways have been implicated in multiple diseases, such as cancers, diabetes, Alzheimer’s disease and COPD [11,28,29]. It is not surprising that the ER stress pathway provides attractive pharmacological targets and studies on this pathway will surely bring valuable information for both basic and therapeutic research. Our studies shed lights on the crosstalk between the oxidative stress signal and the ER stress response, providing new avenues for drug discovery research for COPD and other ER stress related diseases. The identified compounds, characterized with some important features, can certainly prove useful as tools for future studies toward this direction.
